# Structural Centrality Versus Inferred Ancestral State: Unraveling Hidden Heterogeneity in Large HIV Molecular Clusters

**DOI:** 10.3390/v18101136

**Published:** 2026-10-14

**Authors:** Lingxuan Lai, Yefei Luo, Shunming Li, Yingying Song, Hao Wu, Yanshan Cai, Zhenming Tian, Ying Yi, Zhongjin Yao, Huanchang Yan, Zhigang Han, Yu Liu

**Affiliations:** 1 School of Public Health and Management, Guangzhou University of Chinese Medicine, Guangzhou 510006, China; kylin1640@yeah.net (L.L.); 17826565541@163.com (Y.S.); zmtian@gzucm.edu.cn (Z.T.); shandongyy@gzucm.edu.cn (Y.Y.); yaozhongjin@126.com (Z.Y.); 2 Department of AIDS Control and Prevention, Guangzhou Center for Disease Control and Prevention, Guangzhou 510440, China; yefeiluo@foxmail.com (Y.L.); gzcdc_lism@gz.gov.cn (S.L.); gzcdc_wuh@gz.gov.cn (H.W.); caiyanshan1971@163.com (Y.C.); 3 Department of Medical Statistics, School of Public Health, Sun Yat-sen University, Guangzhou 510080, China

**Keywords:** HIV, molecular transmission network, giant component, community detection, bridging members

## Abstract

Large HIV molecular clusters are often treated as homogeneous units, obscuring internal heterogeneity and the distinction between contemporary transmission hubs and inferred ancestral states. Using 935 CRF55_01B *pol* sequences (2008–2020, Guangzhou), we constructed a Tamura-Nei 93 molecular network. The 110-member giant component (GC) was partitioned via Louvain, and bridging members were identified. Phylogenetic analysis and PastML ancestral state reconstruction were performed, with sensitivity analysis at an alternative genetic distance threshold. Louvain identified 11 communities with a core-periphery architecture. Community 1 (C1) served as the dominant structural hub, driven by 21 bridging members who accounted for 82.5% of inter-community links and exhibited significantly higher degree centrality and shorter average shortest path length than non-bridging members (*p* < 0.05). However, ancestral state reconstruction most frequently assigned the root state to Community 2, revealing a striking discordance between structural centrality and inferred ancestry. At the 0.008 threshold, the GC expanded substantially, yet C1 persistently remained the primary hub, preserving this decoupling. Community-level analysis unravels hidden heterogeneity and decouples structural centrality from inferred ancestral state. Bridging-member identification highlights structurally important individuals for enhanced surveillance and further evaluation.

## 1. Introduction

Large and growing clusters identified through longitudinal HIV molecular surveillance can encompass substantial numbers of people living with HIV (PLWH) and may contribute disproportionately to ongoing local transmission [1,2,3,4,5,6,7]. These clusters are characterized by superspreading events, densely connected transmission chains, and critical bridging nodes that link distinct subgroups [8,9,10,11,12]. Their size and structural complexity can make component-wide prioritization difficult, underscoring the need to identify the substructures and members that contribute most to network connectivity [6,13,14].

The internal structure of molecular transmission networks is increasingly recognized as a guide for refined interventions, moving beyond the assumption that all members of a large cluster contribute equally to network connectivity [6,8,11,14,15]. In large clusters, super-spreaders appear as nodes with high degree centrality that drive local spread through dense connections; dense subclusters may indicate focal transmission activity; and bridging nodes linking distinct subclusters connect otherwise separate network communities [6,11,12].

To characterize these topological features, community detection algorithms, exemplified by Louvain [16,17], Infomap [18], and stochastic block models [19], provide a network-science framework for resolving internal structure within large connected components [20]. These methods do not replace the genetic distance (GD) threshold used to construct molecular networks; instead, they further partition connected components into internally cohesive network communities [20,21]. They accurately localize bridge individuals and putative super-spreaders and facilitate dynamic surveillance of cluster growth trajectories [5,6,11,12,22,23,24]. The resulting metrics furnish measurable and actionable intervention endpoints, which can be directly prioritized in public health planning [6,11,25]. Consequently, these approaches can support more focused interventions in large network settings by directing attention to structurally pivotal subgroups rather than treating the entire cluster as a homogeneous unit [6,7].

In this study, we focused on the giant component (GC) of the CRF55_01B molecular network in Guangzhou and used community partitioning to identify structurally important subgroups that may inform enhanced molecular surveillance and targeted public health response.

## 2. Materials and Methods

### 2.1. Study Population and GC Identification

This study included newly diagnosed, treatment-naïve HIV-positive adults in Guangzhou, China, from January 2008 to December 2020, from whom HIV-1 *pol* gene sequences were obtained through routine molecular surveillance. Demographic and behavioral data, sample collection, gene amplification, sequencing, and genotyping were performed as described in our previous pipeline [4]. All participants provided written informed consent, and the study was approved by the Institutional Review Board of School of Public Health, Sun Yat-sen University (No. 2023073). Only individuals with confirmed CRF55_01B infection were retained for analysis.

For the CRF55_01B molecular transmission network, sequences were aligned using MAFFT v7 [26], and pairwise GD were calculated using the Tamura-Nei 93 (TN93) model. To determine the CRF55_01B-specific GD threshold, we evaluated thresholds from 0.001 to 0.015 substitutions/site in increments of 0.001. Following an established data-driven approach [27] and our previous Guangzhou molecular network analysis [4,28], we selected the minimum GD threshold yielding the maximum number of identifiable clusters. This criterion identified 0.005 substitutions/site for the primary analysis (Figure S1). The molecular network was constructed using HIV-TRACE v0.9.1 [29], following the previously described workflow [27]. Individuals were represented as nodes, with undirected edges connecting sequence pairs with GD ≤ 0.005 substitutions/site. Molecular clusters were defined as connected components containing at least two nodes, and the largest connected component was defined as the GC. Visualization and topological analyses were conducted in Cytoscape 3.7.0 [30].

### 2.2. Community Partition and Algorithmic Sensitivity Analysis Within the GC

We used the Louvain method [16,17] to partition the GC into densely connected communities by maximizing modularity [31]. Louvain was selected because we aimed to characterize modular organization within a fixed, unweighted GD network and to subsequently quantify inter-community connectivity and bridging members based on the resulting partition. The analysis was implemented using the cluster_louvain () function of the R igraph v2.1.4 package [32]. To reduce stochastic variation, Louvain partitioning was repeated 100 times using integer seeds 1–100 at a resolution of 1.0, and the partition with the highest modularity (*Q*) was retained for subsequent analyses.

We additionally applied Leiden [33] and Infomap [18] to the same GC as algorithm sensitivity analyses. Agreement between the resulting partitions and the Louvain partition was quantified using the adjusted Rand index (ARI) [34]. The number and composition of detected communities, the identity of bridging members, and the location of the principal cross-community hub across the three algorithms were further compared.

### 2.3. Centrality Profiling and Bridging Connectivity of the GC

#### 2.3.1. Structural and Centrality Metrics

To quantify the internal cohesiveness of each community and the positional importance of PLWH within the network architecture, we computed both community-level structural metrics and nodal-level centrality metrics. At the community level, network density and average shortest path length (ASPL) were calculated within each community-induced subgraph. Network density was defined as the proportion of observed intra-community links among all possible ties, reflecting subcluster tightness. ASPL was defined as the mean geodesic distance between all pairs of reachable nodes, indicating information-flow efficiency. At the nodal level, four canonical centrality measures were evaluated [35]: degree centrality (the number of direct connections), betweenness centrality (the fraction of all-pairs shortest paths traversing a node), closeness centrality (the reciprocal of the mean shortest-path distance to all other reachable nodes), and eigenvector centrality (which assigns higher scores to nodes connected to other highly central nodes). In addition, nodal-level ASPL (the mean shortest-path distance from a node to all other reachable nodes) was also calculated.

To enable cross-community comparisons while mitigating the influence of community size, degree centrality was normalized by dividing by *n* − 1, with *n* representing the community size. Closeness and betweenness centralities were computed via the normalized implementations in the R igraph package, and eigenvector centrality was scaled accordingly. Notably, for comparisons between bridging and non-bridging members (see below) at both the within-community and complete-GC scales, raw (unnormalized) nodal-level degree centrality, betweenness centrality, and ASPL were used to directly reflect the absolute connectivity and positional differences in these key nodes.

#### 2.3.2. Bridging Link Categorization and Bridging Capacity Quantification

Following community assignment, GC links were categorized as intra-community or inter-community links [6]. Individuals incident to at least one inter-community link were operationally defined as bridging members. For each community, we quantified the number and proportion of bridging members, the number of inter-community links, and the number of distinct connected external communities. The structural hub was operationally defined as the community with the largest number of observed inter-community links. Bridging-member counts and the number of connected external communities were additionally reported to characterize its cross-community connectivity. A member-level heatmap summarized inter-community links from bridging members in this hub to individual external communities.

#### 2.3.3. Multi-Scale Comparative Analysis

Comparative analyses were conducted at three scales. First, network metrics were compared across communities to characterize structural heterogeneity within the GC, including community-level density and ASPL as well as nodal-level normalized centrality profiles. Second, within the primary cross-community hub (the community with the most inter-community links), centrality distributions were compared between bridging and non-bridging members using raw degree, unnormalized betweenness centrality, and ASPL. Third, the same bridging-versus-non-bridging comparison was extended to the complete GC to evaluate structural prominence at the whole-network level.

### 2.4. Phylogenetic Contextualization and Ancestral Inference of the GC

To investigate the relationship between network-derived communities and evolutionary history, and to infer ancestral community states within the GC, maximum-likelihood (ML) phylogenetic analysis was conducted using IQ-TREE v3.0.1 [36]. The best-fit nucleotide substitution model was selected using ModelFinder implemented in IQ-TREE v3.0.1 [37] according to the Bayesian information criterion. Branch support was evaluated using 1000 ultrafast bootstrap (UFBoot) replicates and 1000 Shimodaira–Hasegawa approximate likelihood-ratio test (SH-aLRT) replicates [38,39]. The resulting ML tree was midpoint-rooted for visualization and subsequent ancestral state reconstruction.

For ancestral state reconstruction, community assignments derived from the molecular network were encoded as discrete categorical traits at the tree tips. Using PastML v1.9.34 [40], the marginal posterior probabilities approximation (MPPA) method was applied under the Felsenstein 1981 (F81) substitution model to infer the ancestral states of internal nodes. F81 was selected as a parsimonious model because parameter-rich transition models are difficult to estimate reliably from a single multistate trait [40]. Inferred state changes were therefore interpreted descriptively rather than as epidemiological transition rates. This reconstruction was intended for phylogenetic contextualization, not independent validation of the community partition. The annotated tree was visualized using the R ggtree v3.14.0 package [41].

To place the GC communities within the broader CRF55_01B phylogenetic context, an ML phylogeny was also reconstructed using all 935 CRF55_01B sequences following the same IQ-TREE workflow. Ancestral states were reconstructed using the same PastML procedure.

Finally, to compare network communities with phylogeny-based clustering, the GC ML phylogeny was additionally analyzed using ClusterPicker v1.2.3 [42] and TreeCluster v1.0.5 [43]. ClusterPicker was applied with a minimum UFBoot support threshold of 95% and a maximum within-cluster *p*-distance of 0.005, whereas TreeCluster was applied using both the max_clade mode, which imposes a clade constraint, and the max mode, which does not, with a tree-path distance threshold of 0.005. Non-singleton phylogeny-based clusters were then compared with Louvain communities according to membership overlap, nesting patterns, and the effect of imposing a clade constraint. To directly compare phylogenetic context with network bridging, Louvain-defined bridging members were also mapped onto the GC ML phylogeny, and their distribution was examined in relation to the PastML-inferred community states.

### 2.5. Sensitivity Analysis Using an Alternative Genetic Distance Threshold

To evaluate the robustness of the principal network findings to the GD threshold, we repeated the molecular network analysis at 0.008 substitutions/site, which was established in our previous Guangzhou-wide HIV-1 molecular network study [4]. At 0.008, we reconstructed the network for all 935 CRF55_01B sequences, identified the new GC, and reapplied the Louvain algorithm using the same workflow as in the primary analysis. To facilitate cross-threshold comparison, communities at 0.008 were relabeled according to the dominant membership flow from the primary partition: communities without a clear predecessor received new labels; no one-to-one correspondence between partitions was imposed. We then compared network size, modularity, community organization, intra- and inter-community connectivity, and bridging patterns.

Bridging members at 0.008 were identified using the same operational definition as in the primary analysis, and bridging characteristics were summarized using the same measures. To assess whether bridging members retained distinctive structural positions, we compared bridging and non-bridging members at two scales: across the complete GC and within the primary cross-community hub, using raw (unnormalized) degree centrality, unnormalized betweenness centrality, and ASPL. Group differences were assessed using the Wilcoxon rank-sum test.

For phylogenetic sensitivity analysis, the ML tree of all 935 CRF55_01B sequences was pruned to the 337 members of the 0.008 GC. The final community labels were encoded as tip states, ancestral states were reconstructed in PastML using the MPPA method under the F81 model, and the tree was midpoint-rooted.

### 2.6. Statistical Analysis

Categorical variables were compared using Pearson’s chi-square test or Fisher’s exact test, as appropriate, with missing observations treated as a separate category. Continuous network metrics were compared using the Wilcoxon rank-sum test for two-group comparisons and the Kruskal–Wallis test for comparisons across communities. All tests were two-sided, and *p* < 0.05 was considered statistically significant. Statistical analyses were performed in R v4.4.2.

## 3. Results

### 3.1. Characteristics of the CRF55_01B Epidemic and the GC

**Figure 1:**
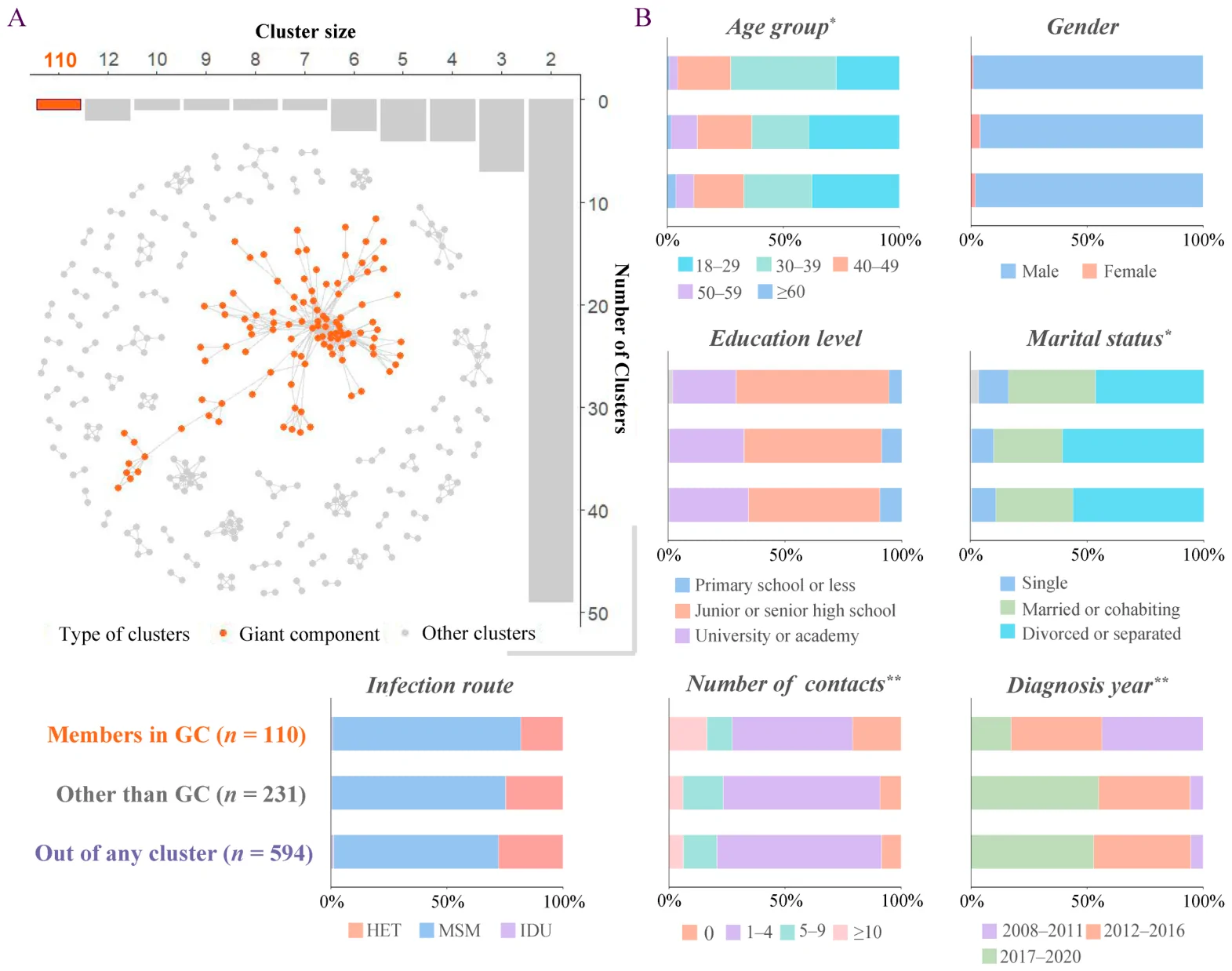
Overall structure of the CRF55_01B molecular transmission network and demographic and epidemiological characteristics according to network position. (**A**) Molecular cluster-size distribution and network visualization at a TN93 genetic distance threshold of 0.005 substitutions/site. Bars show the number of clusters at each observed cluster size; the 110-member GC is shown in orange and the remaining molecular clusters in gray. Nodes represent individuals and links represent pairwise genetic links. The median cluster size was 2 ± 1 (median ± IQR). (**B**) Distributions of age group, gender, education level, marital status, infection route, number of named contacts, and diagnosis year among members of the GC (*n* = 110), members of other molecular clusters (*n* = 231), and individuals outside any cluster (*n* = 594). Asterisks indicate significant overall differences across the three groups (* *p* < 0.05; ** *p* < 0.001). Abbreviations: TN93—Tamura-Nei 93; GC—giant component; IQR, interquartile range; HET—heterosexual contact; IDU—injection drug use; MSM—men who have sex with men.

From 2008 to 2020, molecular surveillance in Guangzhou identified 935 PLWH infected with CRF55_01B. Using a GD threshold of 0.005 substitutions/site (Figure S1), 341 (36.5%) sequences formed 74 clusters comprising 652 links, with cluster sizes ranging from 2 to 110 members. Most clusters were small (≤4 members; 60/74, 81.1%). The largest cluster comprised 110 members (32.3% of 341) and 379 links (58.1% of 652) and was designated as the GC (Figure 1A).

Members of the GC exhibited distinct demographic and epidemiological profiles compared with those in non-GC clusters or outside any cluster. PLWH aged 30–39 years (45.5% vs. 24.7% vs. 29.3%, respectively) and those diagnosed during 2008–2011 (43.6% vs. 5.6% vs. 5.4%) were overrepresented in the GC. Conversely, recent diagnoses during 2017–2020 were more common in the two non-GC groups (17.3% vs. 55.0% vs. 52.9%). The GC also contained higher proportions of individuals reporting either no named contacts (20.9% vs. 9.1% vs. 8.4%) or ≥10 contacts (16.4% vs. 6.1% vs. 6.2%) (Figure 1B; Table S1).

### 3.2. Community Partition Reveals Core-Periphery Structure and Heterogeneous Demographic Profiles

**Figure 2:**
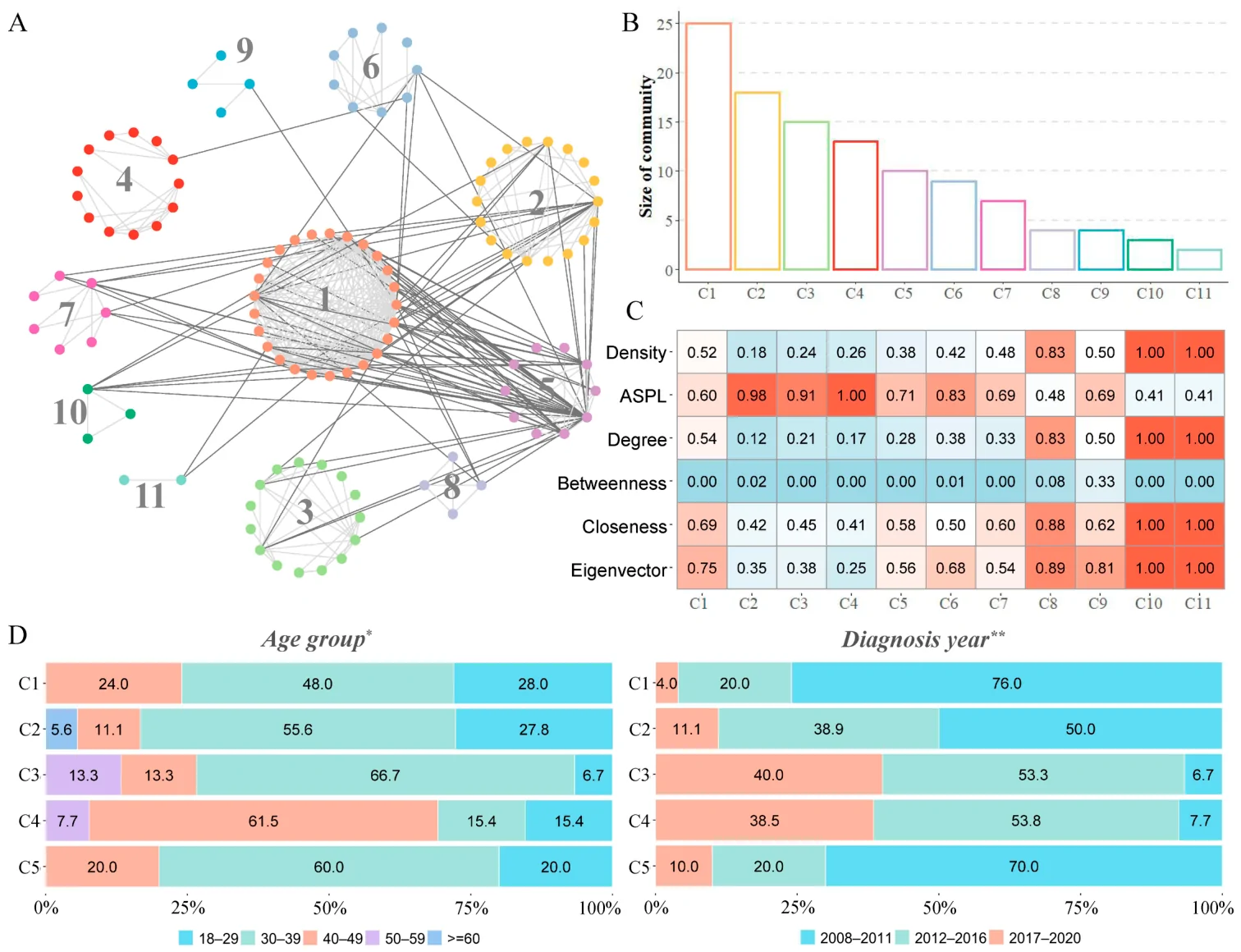
Louvain community structure, structural characteristics, and demographic profiles within the CRF55_01B GC. (**A**) Louvain partition of the 110-member GC into 11 communities (C1–C11). Nodes represent individuals, links represent pairwise genetic links, node colors indicate community assignment, and dark and light links indicate inter- and intra-community connections, respectively. Node and community positions in the displayed core–periphery (“satellite”) layout were manually adjusted after Louvain community detection for visual clarity only; this adjustment did not alter community assignments, nodes, edges, edge weights, or any analytical or statistical results. (**B**) Sizes of the 11 communities. (**C**) Heatmap of network density, median ASPL scaled to the largest community-level median (2.42), and median normalized degree, betweenness, closeness, and eigenvector centralities across the 11 community-induced subgraphs. In panel C, blue and red indicate lower and higher values, respectively. (**D**) Age-group and diagnosis-year distributions across the five major communities (C1–C5); values are within-community percentages, and asterisks indicate significant overall differences (* *p* < 0.05; ** *p* < 0.001). Abbreviations: GC—giant component; ASPL—average shortest path length.

Louvain partitioning of the GC identified 11 distinct communities (*Q* = 0.442), designated C1–C11 (Figure 2A). This partition exhibited a pronounced core-periphery architecture. C1, which contained the largest number of nodes, occupied the topological core and maintained extensive inter-community connections. In contrast, the other communities (C2–C11) functioned as peripheral clusters with relatively sparse connectivity among themselves.

Community sizes varied widely, ranging from 2 to 25 members (Figure 2B). Figure 2C summarizes network density, maximum-scaled median ASPL, and normalized nodal centrality profiles across all 11 communities. Because normalized network indices can be unstable in very small communities, interpretation focused on C1–C7, each containing at least five members. Within this subset, C1 had the highest network density (0.52), the lowest ASPL, and the highest median normalized degree (0.54), closeness (0.69), and eigenvector centrality (0.75). Its median normalized betweenness centrality was 0.00, indicating high local connectivity without disproportionately occupying shortest paths between other nodes. C2–C7 generally showed sparser connectivity and lower centrality profiles (Figure 2C; Table S2).

Beyond topology, the communities exhibited significant demographic heterogeneity. Given that the five largest communities (C1–C5, each ≥10 members) encompassed 73.6% (81/110) of the GC, demographic comparisons focused on this subset. C1 and C5 were dominated by early diagnoses (2008–2011: 76.0% and 70.0%, respectively), whereas C3 and C4 had higher proportions of recent diagnoses (2017–2020: 40.0% and 38.5%, respectively). Age distributions also differed significantly (*p* = 0.035); C4 had the oldest profile, with 61.5% of members aged 40–49 years, whereas C1–C3 and C5 were predominantly composed of individuals aged 30–39 years (48.0%, 55.6%, 66.7%, and 60.0%, respectively; Figure 2D).

### 3.3. Bridging Members in C1 Exhibit High Centrality and Drive Cross-Community Connectivity

**Figure 3:**
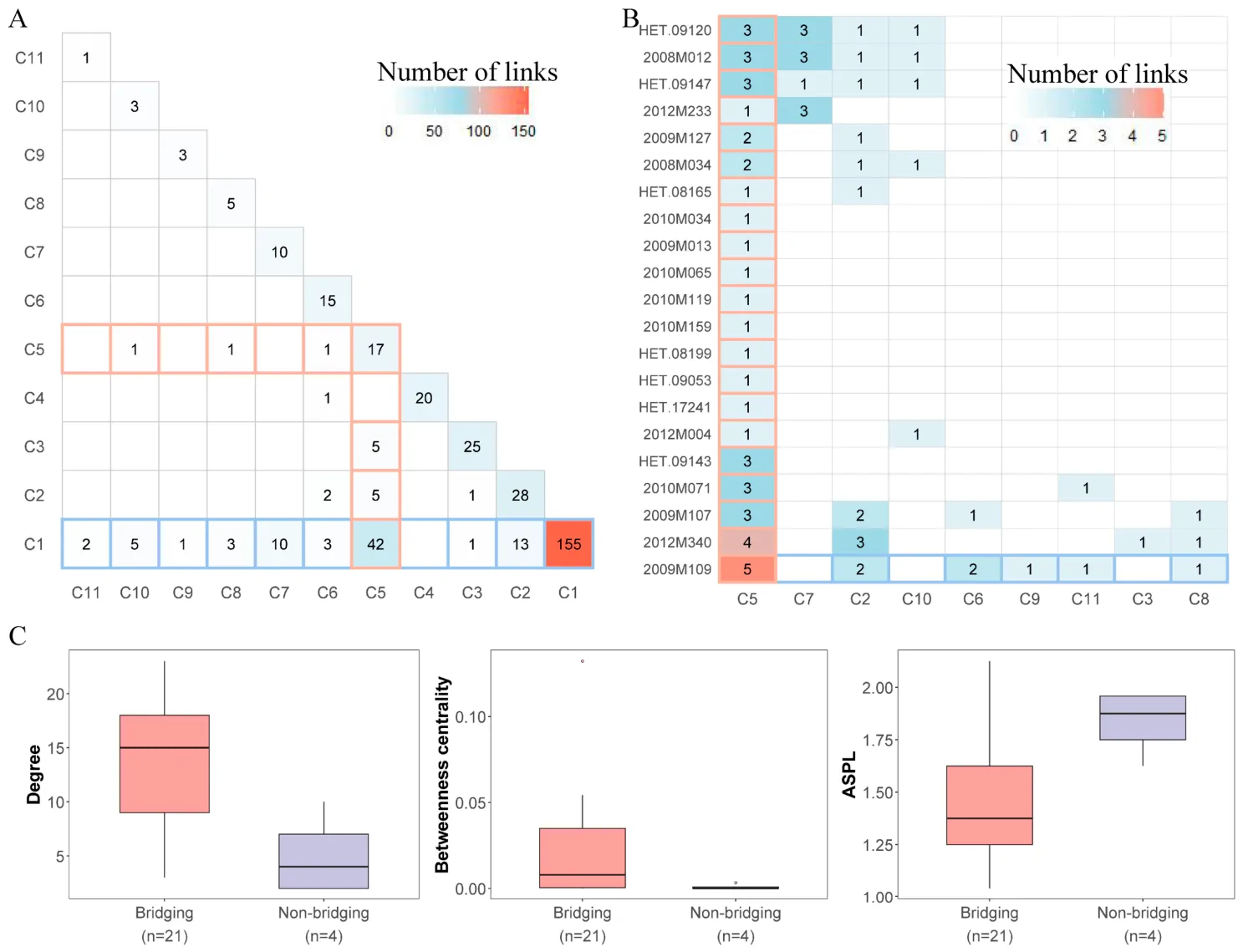
Inter-community connectivity and structural characteristics of bridging members in Community 1 within the CRF55_01B GC. (**A**) Matrix of intra-community and inter-community links among the 11 communities. Diagonal cells show intra-community links, off-diagonal cells show inter-community links, and cell values indicate the number of links. Intra- and inter-community links involving Community 1 and Community 5 are highlighted in blue and orange, respectively. (**B**) Heatmap showing the number of cross-community links from each bridging member in Community 1 to members of Communities 2–11. Each row represents a bridging member in Community 1, columns represent externally connected communities, and cell color indicates the number of links. Community 5, the community linked to every bridging member of Community 1, is highlighted in orange. Within Community 1, the bridging member that connects to the largest number of communities is highlighted in blue. (**C**) Boxplots comparing raw degree, unnormalized betweenness centrality, and ASPL between bridging members (*n* = 21) and non-bridging members (*n* = 4) within Community 1. Group differences were assessed using the Wilcoxon rank-sum test. Abbreviations: GC—giant component; ASPL—average shortest path length.

Inter-community connectivity was highly centralized around C1 and its peripheral counterparts (C2–C11; Figure 3A). C1 connected to nine external communities except C4 and accounted for 82.5% (80/97) of all inter-community links. The strongest pairwise connection was observed between C1 and C5, comprising 42 links. C1 also contained the largest number of bridging members (*n* = 21), whose cross-community connections were heterogeneously distributed (Figure 3B). Notably, all 21 bridging members were connected to C5, while some also connected to other communities.

Centrality profiling showed that these bridging members occupied structurally prominent positions within C1. Compared with non-bridging members, bridging members had a higher raw degree (median ± IQR, 15 ± 9 vs. 4 ± 5; *p* = 0.017), higher unnormalized betweenness centrality (*p* = 0.059), and a shorter ASPL (1.375 ± 0.375 vs. 1.875 ± 0.208; *p* = 0.019; Figure 3C).

To assess the robustness of the principal structural findings to the choice of community-detection algorithm, the Louvain partition was compared with those obtained using Leiden and Infomap (Table S4). Leiden reproduced the Louvain partition exactly (ARI = 1.000). In contrast, Infomap identified 13 communities (ARI = 0.676) and 41 bridging members, 34 of whom overlapped with the Louvain-defined set. Notably, the 36-member principal hub identified by Infomap encompassed all 25 members of Louvain C1 (Figure S2A,B). These findings indicate that the prominence of the C1-associated network region was preserved across algorithms, although community boundaries and individual bridging-member assignment varied. Most available demographic and epidemiological characteristics were similar between bridging and non-bridging members, although diagnosis year differed significantly between the groups (Table S5).

### 3.4. Phylogenetic Analysis Highlights the Discordance Between Network Structure and Inferred Ancestral State

**Figure 4:**
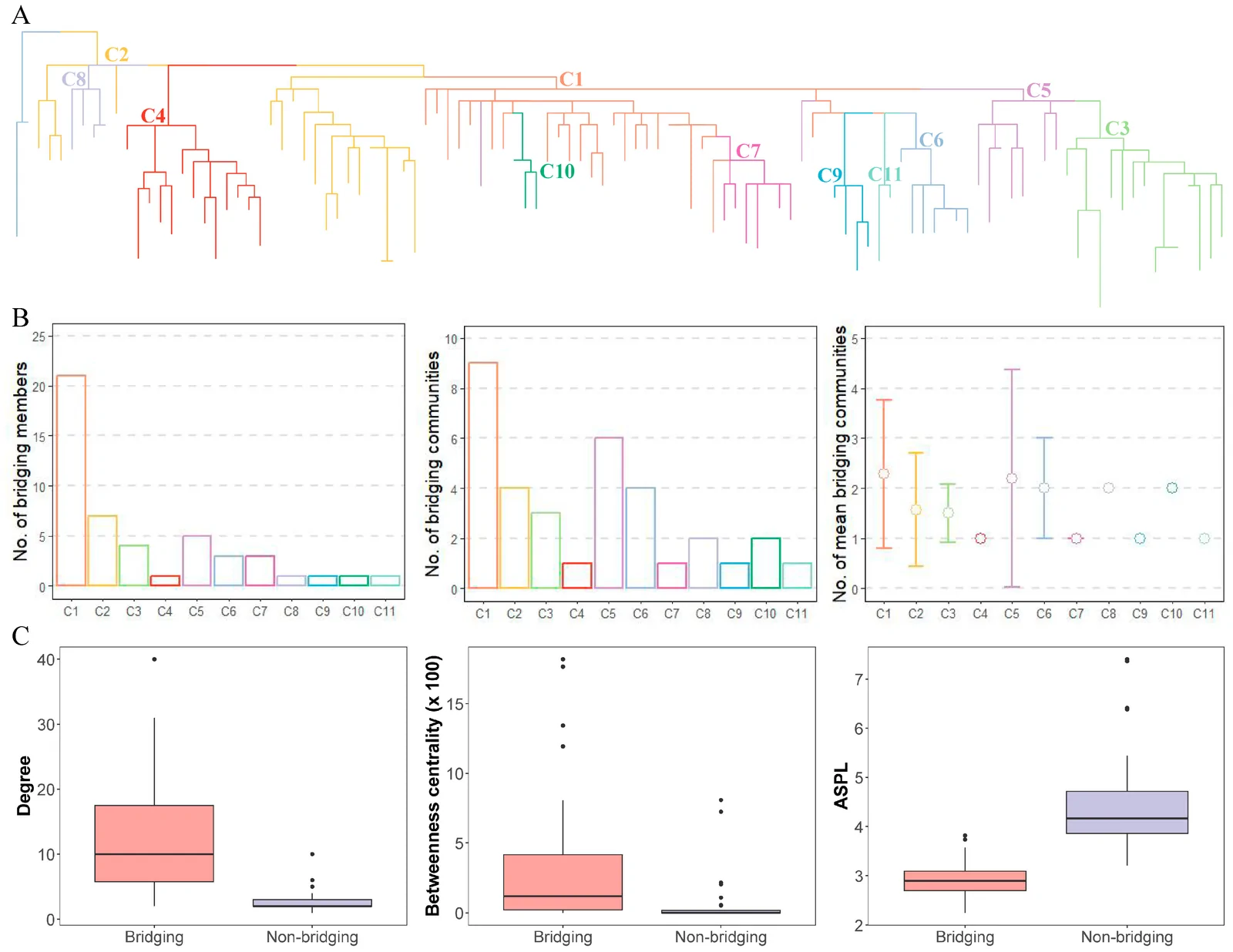
Phylogenetic context and community-level bridging architecture of the CRF55_01B GC. (**A**) Maximum-likelihood phylogenetic tree of the 110 CRF55_01B sequences in the GC. Branch colors indicate observed or PastML-reconstructed states for the 11 network communities; gray branches indicate unresolved ancestral states. (**B**) Community-level bridging profiles across C1–C11: number of bridging members, number of distinct external communities bridged, and mean number of external communities connected per bridging member (mean ± SD). Error bars are omitted for communities with a single bridging member. (**C**) Comparisons of raw degree, unnormalized betweenness centrality (displayed in units of 100), and ASPL between bridging members (*n* = 48) and non-bridging members (*n* = 62) across the complete GC. Bridging members were incident to at least one inter-community link; group differences were assessed using the Wilcoxon rank-sum test. Abbreviations: GC—giant component; SD—standard deviation; ASPL—average shortest path length.

To examine the relationship between network-defined communities and phylogenetic structure, we performed ML phylogenetic analysis on the 110 CRF55_01B sequences within the GC (see Section 2). Members of the same Louvain communities showed recognizable phylogenetic grouping (Figure 4A). Complementary phylogeny-based clustering identified 15 non-singleton ClusterPicker clades (33 sequences), 24 clade-constrained TreeCluster clusters (52 sequences), and 26 TreeCluster groups without the clade constraint (57 sequences; Figure S3). Removing the clade constraint modestly increased sequence coverage, but substantial numbers of sequences remained singletons (77, 58, and 53, respectively). Thus, phylogeny-based clustering resolved more localized subsets rather than reproducing the complete 11-community Louvain partition. However, PastML ancestral state reconstruction assigned the midpoint-defined root state most frequently to C2 (posterior probability = 0.698), whereas C1 had the strongest cross-community connectivity. Mapping the 48 bridging members onto the GC ML phylogeny showed that they were distributed across multiple phylogenetic regions rather than forming a discrete phylogenetic subgroup (Figure S4).

This distinction between structural centrality and inferred ancestral state was further contextualized by community-level bridging profiles (Figure 4B). C1 contained the largest number of bridging members (*n* = 21), connected to nine external communities, and had the highest mean number of external communities connected per bridging member (2.29; SD, 1.49). C5 showed the next broadest reach, connecting to six external communities (mean per bridging member, 2.20; SD, 2.17).

Consistent with their structural role, bridging members across the complete GC (*n* = 48) had higher raw degree and unnormalized betweenness centrality and shorter ASPL than non-bridging members (*n* = 62); all *p* < 0.001 (Figure 4C). Complementary phylogenetic analysis of all 935 CRF55_01B sequences further showed that the five major communities were embedded within the broader CRF55_01B phylogeny rather than forming isolated lineages (Figure S5). Collectively, these findings indicate that network structural centrality and the inferred ancestral state did not coincide: C1 occupied the most prominent cross-community position, whereas C2 had the highest inferred root-state probability.

### 3.5. Threshold-Dependent Network Expansion Preserves C1 as the Primary Structural Hub and Sustains the Decoupling from Inferred Ancestry

At the 0.008 threshold, 563 of 935 (60.2%) sequences formed 67 clusters connected by 3701 links. The GC expanded from 110 to 337 members and contained 3314 links (Figure S6A). All 110 members of the 0.005 GC were retained, whereas 227 additional members were newly incorporated at 0.008. Their reassignment among the 0.008 communities is shown in Figure S6B. Louvain partitioning identified 15 communities (*Q* = 0.301), seven of which contained at least 10 members and together accounted for 89.0% (300/337) of the GC population. Critically, the proportion of inter-community links increased markedly from 25.6% (97/379) at 0.005 to 45.6% (1510/3314) at 0.008, indicating substantially denser cross-community mixing at the higher threshold (Figure S6C; Table S3).

Consistent with the primary analysis, C1 remained the largest community and the primary cross-community hub. It was involved in 969 of 1510 inter-community links (64.2%) and connected to 13 external communities, although its share of inter-community links was lower than the 82.5% observed at 0.005. The increased inter-community mixing at 0.008 was concentrated mainly among C1, C6, C3, and C2 (Figure S6C; Table S3).

At the 0.008 threshold, bridging members also occupied more central structural positions than non-bridging members at both scales. Across the complete GC, bridging members had higher raw degree and unnormalized betweenness centrality and shorter ASPL than non-bridging members (all *p* < 0.001; Figure S7A). Within C1, bridging members had higher raw degree and unnormalized betweenness centrality and shorter ASPL (all *p* < 0.001; Figure S7B).

Phylogenetic analysis of the 337-member GC showed recognizable but incompletely isolated community groupings (Figure S8). PastML reconstructed the midpoint-defined root state as C6 with a posterior probability of 0.9994. Four members assigned to C2 at 0.005 were reassigned to C6 at 0.008; among them, 2008M023 was sampled earliest and had the shortest terminal branch (Figure S8). Because community labels at the two thresholds do not correspond one-to-one, the change from C2 to C6 in the reconstructed root-state assignment is best interpreted as a consequence of threshold-dependent community reassignment. Importantly, C1 remained the primary structural hub, preserving the principal observation that the structural hub did not coincide with the inferred ancestral root state.

## 4. Discussion

This study revealed substantial internal heterogeneity within the GC of the CRF55_01B molecular transmission network in Guangzhou. Community partitioning further resolved the large connected component into substructures with heterogeneous internal and cross-community connectivity, with C1 occupying the dominant structural position. Notably, this structural hub did not coincide with the inferred ancestral root state, which was most frequently assigned to C2. These findings suggest that community-level analysis can provide an intermediate analytical scale between conventional component-level clustering and individual-level centrality, thereby revealing structural heterogeneity that may be obscured when a large connected component is treated as a single unit. Liu et al. similarly demonstrated that community detection can further partition large connected components and resolve epidemiologically relevant heterogeneity within molecular networks [20].

Community detection also provides information that cannot be obtained from node centrality alone. Centrality measures primarily characterize the structural position of individual nodes, whereas community partitioning additionally distinguishes connections occurring within cohesive substructures from those spanning different communities. This distinction enabled us to identify bridging members according to their cross-community connectivity rather than simply selecting individuals with the highest degree. Previous molecular network studies in China have used network positions to identify key nodes or priority intervention targets, including an analysis among MSM in Guangzhou and a densely sampled network study in Shenyang [25,44]. Our findings extend these approaches by showing that potentially important nodes may also be characterized by their role in connecting otherwise differentiated network communities. Nevertheless, network-derived prioritization should not be interpreted as direct evidence of individual transmission or intervention effectiveness. Recent evaluations of molecular HIV surveillance continue to emphasize the need for stronger evidence linking network-informed prioritization to measurable public-health benefit [45].

The discordance between C1 as the structural hub and C2 as the inferred ancestral root state illustrates the different dimensions captured by molecular network and phylogenetic analyses. Genetic distance networks characterize sequence similarity and connectivity under a predefined threshold, whereas phylogenetic trees describe evolutionary relationships among sampled viral sequences. Consistent with this distinction, phylogeny-based clustering did not reproduce the overall Louvain partition, and bridging members were distributed across multiple phylogenetic regions rather than forming a discrete subgroup. These findings indicate that phylogenetic structure provides complementary evolutionary context but does not substitute for network-based identification of cross-community connectivity. Because both analyses were derived from the same sequences, this correspondence should be interpreted descriptively rather than as independent validation. Ancestral state reconstruction is also conditional on the inferred tree topology and rooting scheme; with single consensus pol sequences, it does not establish an epidemiological source [46,47,48]. Thus, our principal observation is that the community dominating contemporary network connectivity need not coincide with the inferred ancestral state, rather than that C2 represents the definitive epidemic origin.

The observed network organization should also be interpreted in the broader context of CRF55_01B transmission. Comparative molecular network analysis among MSM in Guangxi found that CRF55_01B had a particularly high clustering rate, whereas CRF07_BC was more likely to form larger and faster-growing clusters and showed a higher estimated basic reproduction number [49,50,51]. These findings suggest that high clustering propensity and rapid cluster expansion represent related but distinct dimensions of genotype-specific transmission patterns. Sustained CRF55_01B transmission has also been documented outside Guangzhou. In Shenzhen, seven large CRF55_01B clusters were identified, four of which maintained an effective reproduction number above one at the end of follow-up [52]. More recent studies in Henan and Yunnan have likewise identified multiple CRF55_01B transmission clusters, indicating continued geographic dissemination and localized transmission of this recombinant form [53,54]. Together with the present findings, these observations support closer examination of the internal organization of large CRF55_01B clusters rather than relying solely on cluster size or overall clustering rate. Notably, our previous CRF07_BC analysis in Guangzhou also identified a dominant hub, but that hub coincided with the inferred ancestral community [6]. This contrast suggests that community organization and hub dynamics may reflect context-specific transmission patterns rather than fixed genotype-specific characteristics.

Several limitations should be acknowledged. First, molecular links reflect genetic similarity rather than confirmed direct transmission, and network-derived communities are mathematical representations rather than observed social or transmission groups. Second, network topology and community assignment were sensitive to the GD threshold. Increasing the threshold from 0.005 to 0.008 substantially expanded the GC and reorganized community membership, although C1 remained the primary cross-community hub. Such threshold dependence is expected in genetic distance-based molecular networks: recent studies have shown that increasing the GD threshold alters network inclusion and linkage characteristics and that cluster composition may vary substantially across thresholds and genomic regions [55,56]. Community assignments were also partly algorithm-dependent: although the prominence of the C1-associated network region was preserved across algorithms, the assignment of individual bridging members varied. Third, the primary GC comprised only 110 members, and several communities were small. Although repeated Louvain analyses reduced stochastic variation in the selected partition, estimates derived from small communities should be interpreted cautiously. In addition, incomplete sequence sampling can substantially affect HIV cluster detection and reconstructed network topology [57]. Finally, the phylogenetic analyses were based on closely related partial *pol* consensus sequences and midpoint-rooted trees, limiting inference about some internal relationships and the position of the root. Notably, PastML ancestral state reconstruction yields one plausible scenario; alternative models or rooting methods could alter ancestral assignments. Therefore, the inferred root state should not be equated with the true epidemic origin.

## 5. Conclusions

Community-level analysis of large HIV molecular clusters reveals substantial internal heterogeneity and decouples structural centrality from inferred ancestral state. In the CRF55_01B GC, the dominant structural hub did not coincide with the inferred ancestral root state, underscoring the distinction between contemporary network connectivity and model-based phylogenetic inference. Identification of bridging members provides a framework for recognizing structurally important nodes within large molecular clusters and supports future evaluation of network-informed surveillance strategies.

## Data Availability

The data that support the findings of this study are available on request from the corresponding authors. The data are not publicly available due to privacy or ethical restrictions.

## Supplementary Materials

The following supporting information can be downloaded at https://www.mdpi.com/article/10.3390/v18101136/s1, Figure S1: Sensitivity analysis of TN93 GD thresholds for CRF55_01B molecular network construction; Figure S2: Comparison of Louvain and Infomap community assignments in the 0.005 GC; Figure S3: Comparison of ClusterPicker and TreeCluster phylogenetic clustering with Louvain communities in the 110-tip GC; Figure S4: Mapping of 48 Louvain-defined bridging members on the 110-tip GC ML phylogeny; Figure S5: Maximum-likelihood phylogenetic tree of all 935 CRF55_01B sequences, highlighting the five major communities of the 0.005 GC (C1–C5; *n* = 81); Figure S6: Network structure and community reorganization at the 0.008 genetic distance threshold; Figure S7: Structural characteristics of bridging and non-bridging members at the 0.008 genetic distance threshold; Figure S8: Threshold-dependent reassignment of four original C2 members in the 337-sequence phylogeny; Table S1: Demographic and epidemiological characteristics of individuals with CRF55_01B infection according to molecular network position; Table S2: Node-level centrality profiles across the 11 Louvain communities within the CRF55_01B GC; Table S3: Structural and bridging characteristics of the 15 communities in the 0.008 sensitivity network; Table S4: Comparison of Louvain, Leiden, and Infomap community partitions in the 0.005 CRF55_01B giant component; Table S5: Characteristics of bridging and non-bridging members in the CRF55_01B giant component.
