# Supplementary material for: Structural Centrality Versus Inferred Ancestral State: Unraveling Hidden Heterogeneity in Large HIV Molecular Clusters
Source: Viruses. 2026 Oct 14;18(10):1136. doi: 10.3390/v18101136 (PMC13709836; doi:10.3390/v18101136)
Supplement: Supplementary file 1 [file viruses-18-01136-s001.zip › Supplementary Materials Legends.pdf]

Supplementary figure and table legends

Supplementary figures

Figure S1. Sensitivity analysis of TN93 GD thresholds for CRF55_01B molecular network construction.

Network clustering was evaluated from 0.001 to 0.015 substitutions/site in increments of 0.001. Bars show the number of molecular clusters at each threshold. The threshold of 0.005 substitutions/site yielded the maximum number of clusters (n = 74) and was selected for the primary analysis. Abbreviations: TN93, Tamura-Nei 93; GD, genetic distance.

Figure S2. Comparison of Louvain and Infomap community assignments in the 0.005 GC.

(A) The 110-member network colored by Infomap community assignment, with labels marking the Louvain community positions. (B) Correspondence of individual members between Louvain communities C1–C11 and Infomap communities I1–I13; ribbon widths represent numbers of members. The 36-member principal Infomap hub includes all 25 members of Louvain C1. Abbreviation: GC, giant component.

Figure S3. Comparison of ClusterPicker and TreeCluster phylogenetic clustering with Louvain communities in the 110-tip GC.

Louvain community colors are shown on the tree, and the adjacent tracks indicate ClusterPicker, TreeCluster max_clade, and TreeCluster max group assignments using the clustering settings described in Section 2 of the manuscript. ClusterPicker identified 15 non-singleton clades containing 33 sequences, with 77 singletons. TreeCluster max_clade identified 24 non-singleton clusters containing 52 sequences, with 58 singletons. TreeCluster max identified 26 non-singleton groups containing 57 sequences, with 53 singletons. Abbreviation: GC, giant component.

Figure S4. Mapping of 48 Louvain-defined bridging members on the 110-tip GC ML phylogeny.

Red open circles mark the 48 Louvain-defined bridging members on the maximum-likelihood phylogeny of the 110-member GC. Community colors follow the Louvain community assignments. Abbreviations: GC, giant component; ML, maximum likelihood.

Figure S5. Maximum-likelihood phylogenetic tree of all 935 CRF55_01B sequences, highlighting the five major communities of the 0.005 GC (C1–C5; n = 81).

The remaining 854 tips comprise members of the six smaller GC communities (C6–C11; n = 29), members of other molecular clusters (n = 231), and individuals outside any molecular cluster (n = 594). Branches assigned to C1–C5 are colored; other-community and unresolved ancestral states are shown in gray. Ancestral states were reconstructed using PastML with the MPPA method under the F81 model on a midpoint-rooted tree. The scale bar represents 0.005 substitutions per site. Abbreviations: GC, giant component; MPPA, marginal posterior probabilities approximation; F81, Felsenstein 1981.

Figure S6. Network structure and community reorganization at the 0.008 genetic distance threshold.

(A) Louvain partition of the 337-member GC into 15 communities. Nodes represent individuals, links represent pairwise genetic links, and node colors indicate the 0.008 community assignment. Community labels were assigned according to the dominant membership flow from the 0.005 partition; communities without a clear predecessor received new letter-based labels, and no one-to-one correspondence between the two partitions was imposed. Letters a–i in panel A correspond to communities Ca–Ci. (B) Membership flow of the 110 members of the original 0.005 GC into the 0.008 communities. Flows represent node-ID overlap, and flow colors indicate the source community at 0.005. (C) Matrix of intra-community and inter-community links

among the 15 communities. Diagonal cells show intra-community links, off-diagonal cells show inter-community links, and cell values indicate the number of links. Darker purple indicates more links. Black outlines mark diagonal cells. Abbreviations: GD, genetic distance; GC, giant component.

Figure S7. Structural characteristics of bridging and non-bridging members at the 0.008 genetic distance threshold.

(A) Boxplots comparing raw degree, unnormalized betweenness centrality (displayed on a pseudo-log10 scale), and ASPL between bridging members (n = 176) and non-bridging members (n = 161) across the complete 337-member GC. (B) Corresponding comparisons between bridging members (n = 59) and non-bridging members (n = 24) within C1. Boxes indicate medians and interquartile ranges, and points represent individual members. Orange and gray indicate bridging and non-bridging members, respectively. Group differences were assessed using the Wilcoxon rank-sum test; all $p < 0.001$. Abbreviations: GD, genetic distance; ASPL, average shortest path length; GC, giant component.

Figure S8. Threshold-dependent reassignment of four original C2 members in the 337-sequence phylogeny.

Thick terminal branches highlight four sequences assigned to C2 at 0.005 and to C6 at 0.008; the asterisk marks 2008M023 (sampled in 2008), the shortest-branched of the four. Tip and branch colors indicate the 15 community states or PastML-reconstructed ancestral states at 0.008, with colors for the five major communities matched to their dominant 0.005 counterparts. Gray indicates unresolved states. Ancestral states were inferred using the MPPA method under the F81 model on a midpoint-rooted tree. Abbreviations: MPPA, marginal posterior probabilities approximation; F81, Felsenstein 1981.

Supplementary tables

Table S1. Demographic and epidemiological characteristics of individuals with CRF55_01B infection according to molecular network position.

Values are presented as n (%) unless otherwise indicated. The groups comprise members of the GC (n = 110), members of other molecular clusters (n = 231), and individuals outside any cluster (n = 594), with 935 individuals overall. Percentages use the corresponding column totals. p values represent overall comparisons across the three network-position groups, with missing observations treated as a separate category in the corresponding analysis. Abbreviations: PLWH, people living with HIV; GC, giant component; HET, heterosexual contact; IDU, injection drug use; MSM, men who have sex with men.

Table S2. Node-level centrality profiles across the 11 Louvain communities within the CRF55_01B GC.

Values are presented as median ± IQR for normalized degree, closeness, betweenness, and eigenvector centralities calculated within each community-induced subgraph. Degree centrality was normalized by dividing by $n - 1$, where n is the community size; closeness and betweenness used the normalized implementations in R igraph, and eigenvector centrality was scaled accordingly. p values represent overall comparisons across communities using the Kruskal–Wallis test. Abbreviations: GC, giant component; IQR, interquartile range.

Table S3. Structural and bridging characteristics of the 15 communities in the 0.008 sensitivity network.

Community labels were assigned according to the dominant membership flow from the 0.005 partition; communities without a clear predecessor received new letter-based labels, and no one-to-one correspondence between partitions was imposed. Communities are ordered by size. Network density is calculated as $2m/[n(n - 1)]$, where m is the number of intra-community links and n is the community size. Normalized degree was calculated within each community-induced subgraph; values are median ± IQR. Raw degree was used for bridging-versus-non-bridging comparisons in the manuscript. Bridging members are individuals incident to at least one inter-community link. Each inter-community link contributes to the incident-link count for both endpoint communities. Abbreviation: IQR, interquartile range.

Table S4. Comparison of Louvain, Leiden, and Infomap community partitions in the 0.005 CRF55_01B giant component.

The same undirected, unweighted 110-node, 379-link GC was used for all algorithms. The main hub was the community incident to the largest number of inter-community links. ARI values compare each partition with the Louvain partition. The 36-member Infomap hub included all 25 members of Louvain C1. Infomap optimizes map-equation code length; its modularity is reported descriptively. Community boundaries and bridging status depend on the partition. Abbreviations: GC, giant component; ARI, adjusted Rand index; Q, modularity.

Table S5. Characteristics of bridging and non-bridging members in the CRF55_01B giant component.

Values are presented as n (%) unless otherwise indicated. Percentages use column totals: all GC members (n = 110), bridging members (n = 48), and non-bridging members (n = 62). Missing values were included as categories in the p-value tests. Pearson chi-square or Fisher exact tests were used; sparse tables larger than 2 × 2 used Fisher's Monte Carlo method with 10,000 replicates. Abbreviations: PLWH, people living with HIV; GC, giant component; HET, heterosexual contact; IDU, injection drug use; MSM, men who have sex with men.
